# Supplementary material for: Pre-movement sensorimotor oscillations shape the sense of agency by gating cortical connectivity
Source: Nat Commun. 2025 Apr 16;16:3594. doi: 10.1038/s41467-025-58683-9 (PMC12000325; doi:10.1038/s41467-025-58683-9)
Supplement: Supplementary file 1 — Supplementary Information [file 41467_2025_58683_MOESM1_ESM.pdf]

Supplementary figures

Pre-movement sensorimotor oscillations shape the sense of agency by gating cortical connectivity

Tommaso Bertoni* [1,2], Jean-Paul Noel [3], Marcia Bockbrader [4], Carolina Foglia [1], Sam Colachis [5], Bastien Orset [6], Nathan Evans[6], Bruno Herbelin[6], Ali Rezai [7], Stefano Panzeri [8], Cristina Becchio [2,9], Olaf Blanke‡ [6], Andrea Serino‡ [1]

[1] MySpace Lab, Department of Clinical Neuroscience, University Hospital Lausanne (CHUV), Lausanne, Switzerland

[2] C'MoN, Cognition, Motion and Neuroscience Unit, Fondazione Istituto Italiano di Tecnologia, Genova, Italy

[3] Department of Neuroscience, University of Minnesota, Minneapolis, Minnesota, U.S.A.

[4] Department of Physical Medicine and Rehabilitation, The Ohio State University, Columbus, Ohio, U.S.A

[5] Medical Devices and Neuromodulation, Battelle Memorial Institute, Columbus, Ohio, U.S.A

[6] Neuro-X Institute, Faculty of Life Sciences, Swiss Federal Institute of Technology (EPFL), Lausanne, Switzerland

[7] Rockefeller Neuroscience Institute, West Virginia University, Morgantown, West Virginia, U.S.A.

[8] Institute for Neural Information Processing, Center for Molecular Neurobiology (ZMNH), University Medical Center Hamburg-Eppendorf (UKE), Hamburg, Germany

[9] Department of Neurology, University Medical Center Hamburg-Eppendorf (UKE), Hamburg, Germany

* Corresponding author: Tommaso Bertoni. Email: tommaso.bertoni90@gmail.com

‡ These authors contributed equally to this work

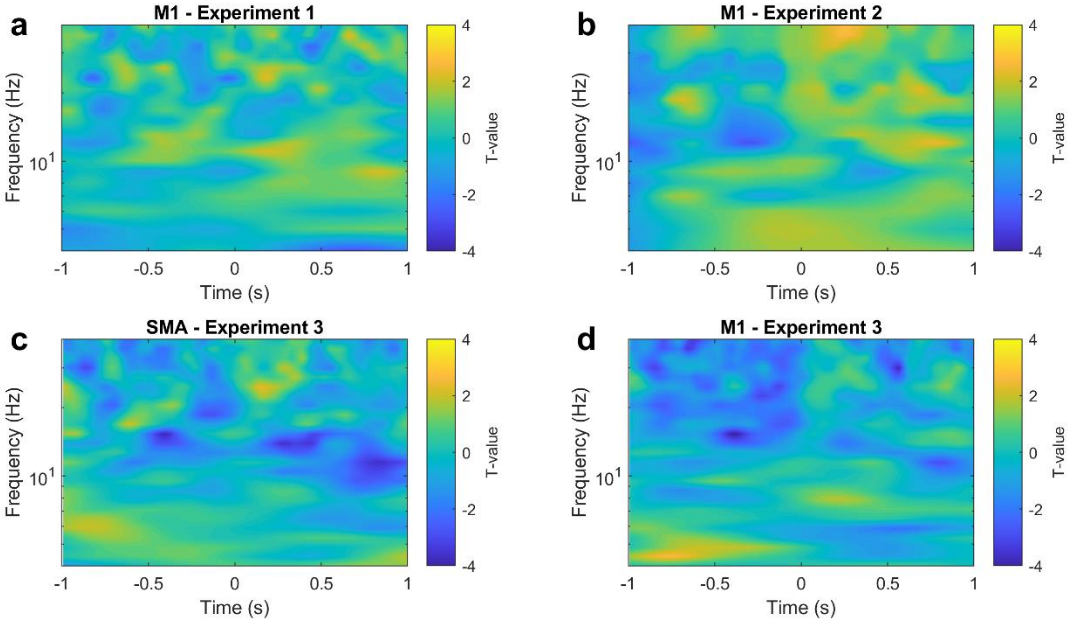

Figure S1. Comparison (t-test, two tailed) of trial-averaged power between high and low agency trials in Experiment 1 (a), 2 (b) and 3 (panel c for SMA, panel d for M1). No significant clusters of power changes were found.

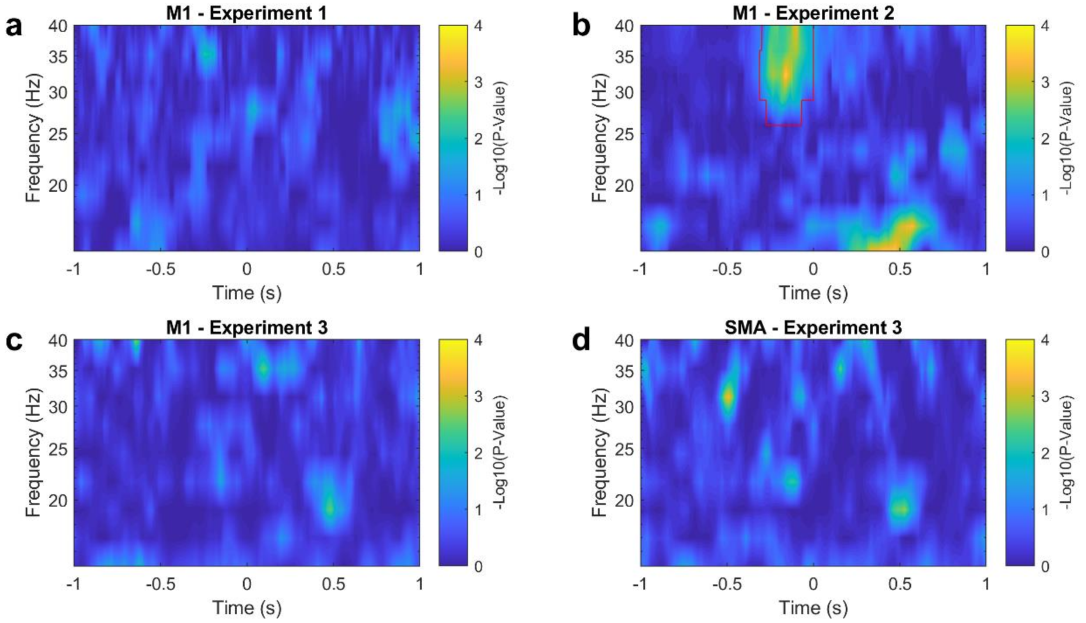

Figure S2. Uncorrected log p-values (comparison with 10000 permutations, one tailed) for phase opposition contrasting high and low agency at higher frequencies, using the same analysis methods reported in the main text. Red contours indicate significant time-frequency regions after cluster correction. Panel (a) refers to Experiment 1, panel (b) to Experiment 2, panel (c) to M1 and panel (d) to SMA in Experiment 3, the two regions showing a significant phase opposition in the alpha range. A significant cluster ($p = 0.0045$) between -0.25/-0.05 s and between 25 and 40 Hz was found in Experiment 2. No significant other clusters of phase opposition were found in the -0.5/0 s time window.

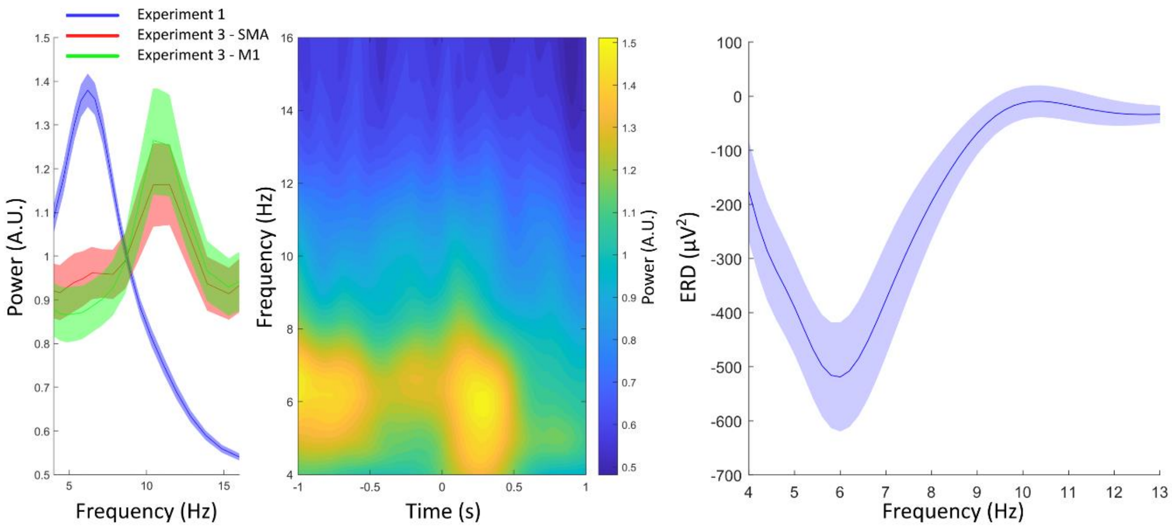

Figure S3. Power spectra comparison between intracranial and EEG data (a) Trial-averaged power spectrum (for Experiment 3 further averaged across subjects) in the baseline period ranging from -2 to -1 seconds from movement for the implanted participant (Experiment 1) and for healthy subjects (Experiment 3, left SMA and M1). For healthy participants, we show data from the left SMA. In the implanted participant, the spectral peak is relatively low in frequency, with the maximal spectral density found at 6 Hz. In healthy participants, the spectral peak is found at the usually reported frequency of 10-12 Hz. The shaded area indicates the standard error. (b) Time-frequency representation of trial-averaged power in Experiment 1. The event-related desynchronization (ERD) is also found around the spectral peak. (c) Trial-averaged ERD in the implanted participant (Experiment 1), computed by subtracting the baseline (-2/-1.5s) average power to the pre-movement (-0.5/0s) average power. The shaded area indicates the standard error.

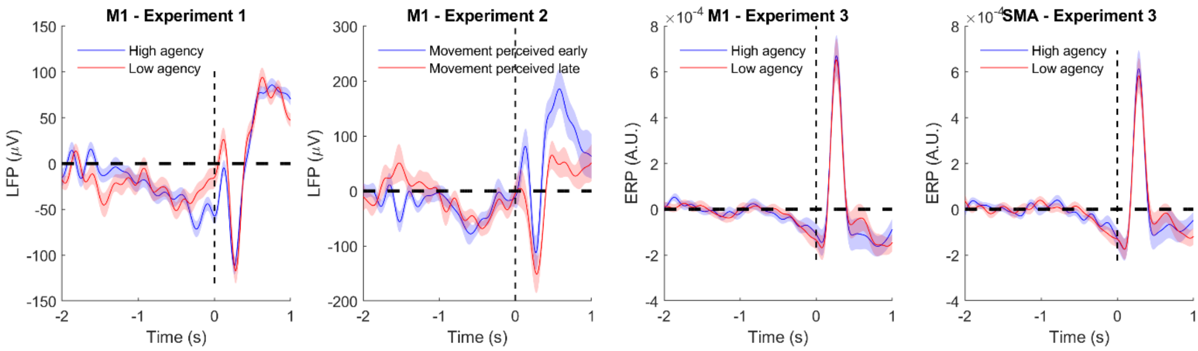

Figure S4. Analysis of readiness potentials. To decouple slow readiness potentials from phase opposition effects, the data was low pass filtered at 5 Hz, and LFPs/ERPs were compared between high and low agency trials. The panels show LFPs/ERPs for Experiment 1, Experiment 2, Experiment 3 – M1, and Experiment 3 – SMA (left to right). Shades indicate standard errors, the horizontal dashed line at 0 is shown to highlight the negative deflection corresponding to the readiness potential, the vertical dashed line indicates the time of the movement. No difference survived multiple comparison correction across timepoints.

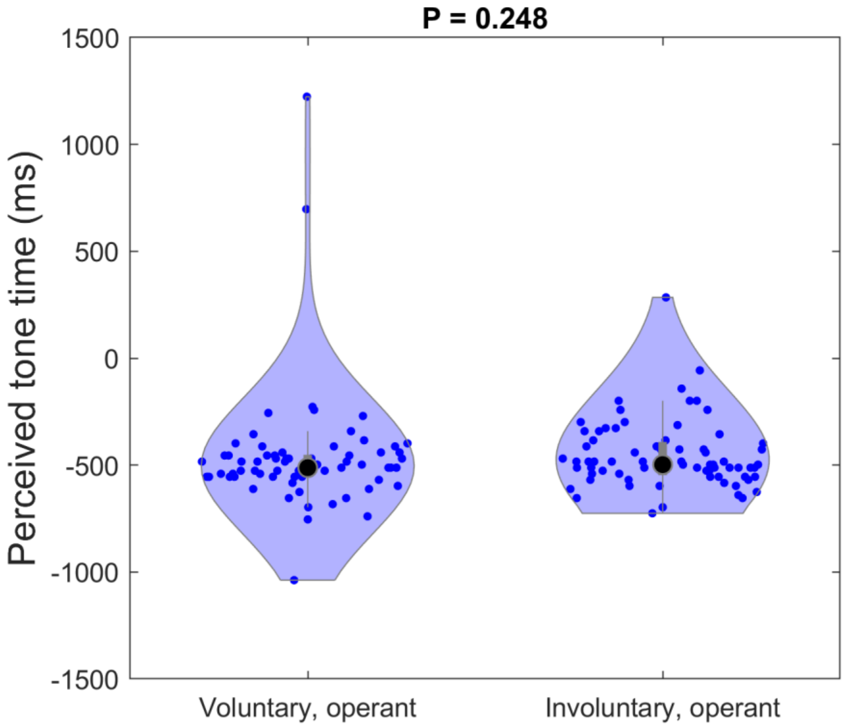

Figure S5. Control analysis on the perceived time of the sound in the operant condition. The p-value reported is from a Wilcoxon rank sum test, as for all other behavioural analyses in Experiment 2.

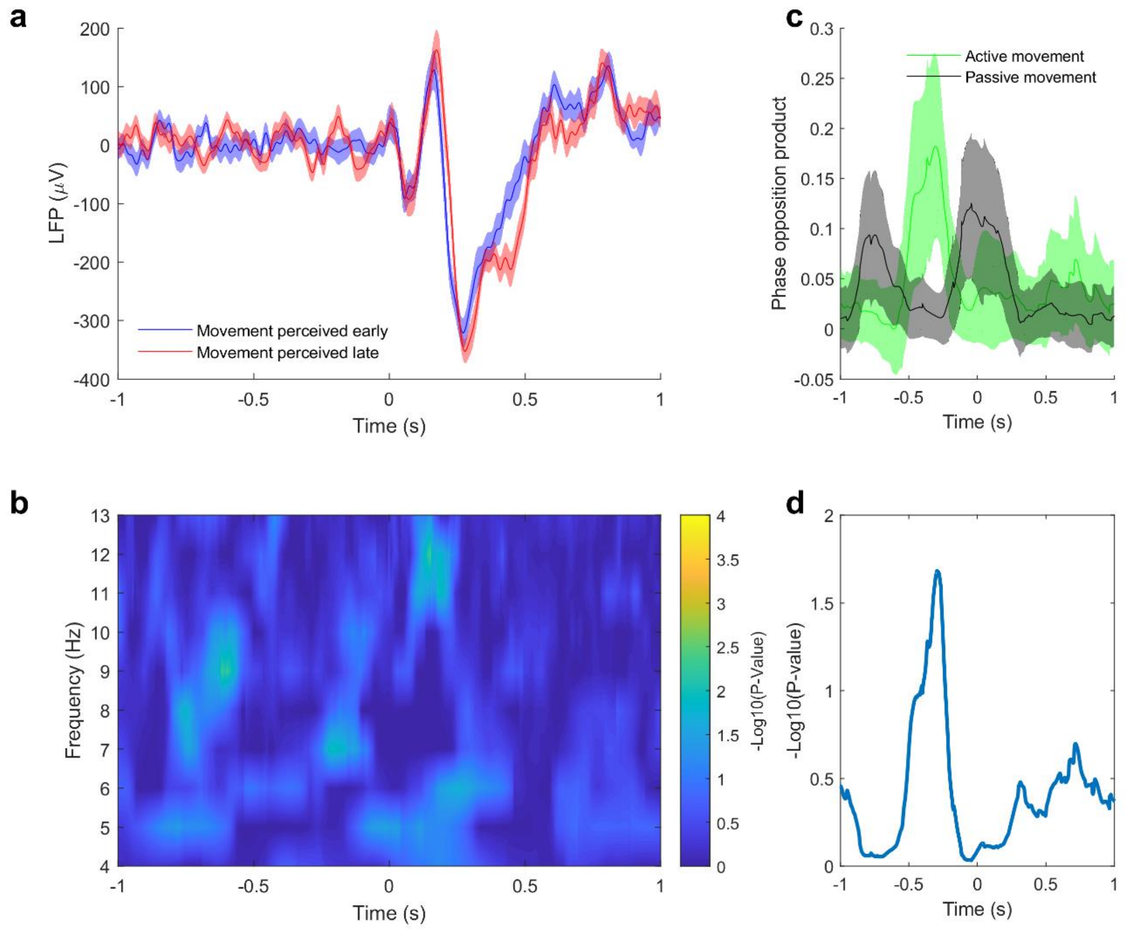

Figure S6. Control analysis on involuntary movement condition. (a) Averaged time course of the LFP for early (red) and late (blue) perception of involuntary movements. Shaded areas indicate standard errors. (b) Uncorrected log p-values for the phase opposition product (comparison with 10000 permutations, one tailed) contrasting early and late movement perception in the involuntary movement condition. No significant cluster emerged after multiple comparison correction. (c) Phase opposition product at 8 Hz between early and late movement perception in the voluntary (green) and involuntary (black) movement condition. Shaded areas indicate 66 % confidence intervals obtained through bootstrapping. (d) Uncorrected log p-values for the comparison shown in panel c. The plot shows the probability that the phase opposition at 8 Hz is larger in the involuntary condition than in the voluntary condition, obtained by counting the number of bootstrapping permutations in which POP(involuntary)>POP(voluntary). The comparison is significant between -341 and -252 ms.

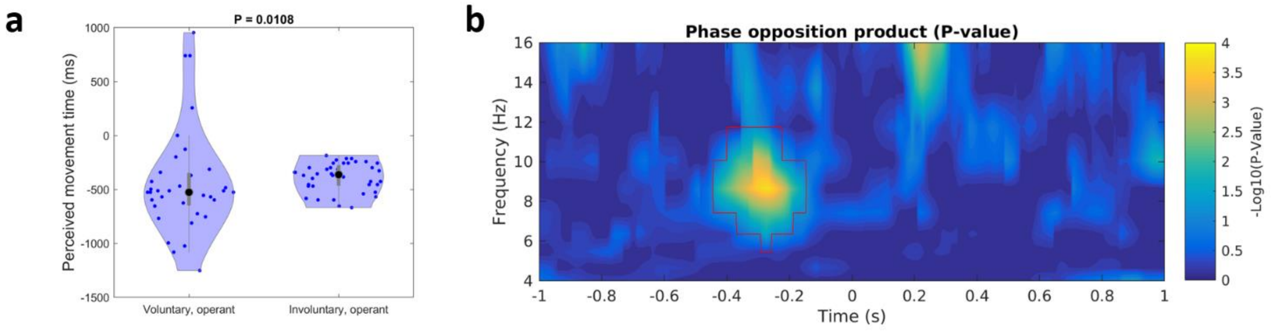

Figure S7. Main results of Experiment 2 for operant trials only. Panel (a) shows the anticipation of perceived movement timing for voluntary vs involuntary movements (p = 0.011, Wilcoxon). Panel (b) shows the phase opposition product for early vs. late perception of the movement within voluntary operant trials. The red contour denotes the significant cluster of phase opposition (p = 0.039).

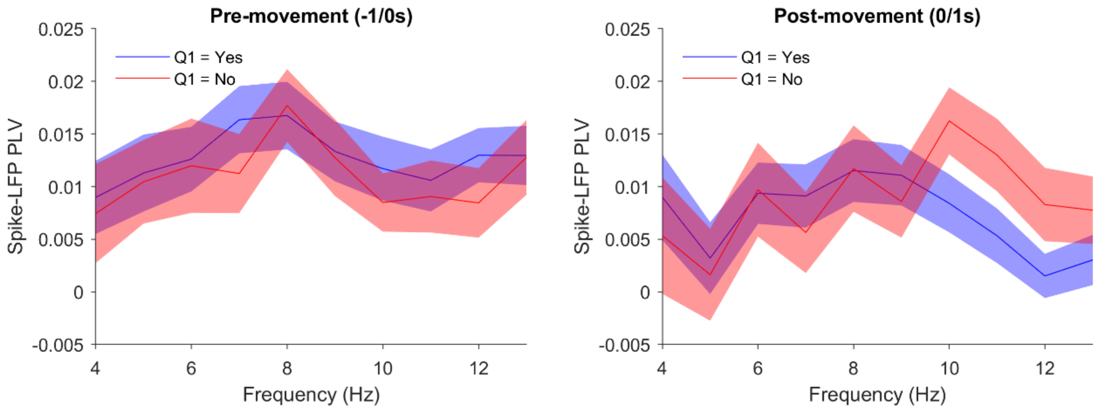

Figure S8. Spike-LFP coupling and agency ratings. We compared PLV values (as in Fig. 5a) between high and low agency trials, in the 1 second before the movement (left plots) and in the 1 second after the movement (right plots). The plots show PLV values with shades indicating 66% confidence intervals obtained by bootstrapping (trials were sampled randomly with replacement 1000 times to estimate the variability of PLV values). No difference survived multiple comparison correction across frequencies.

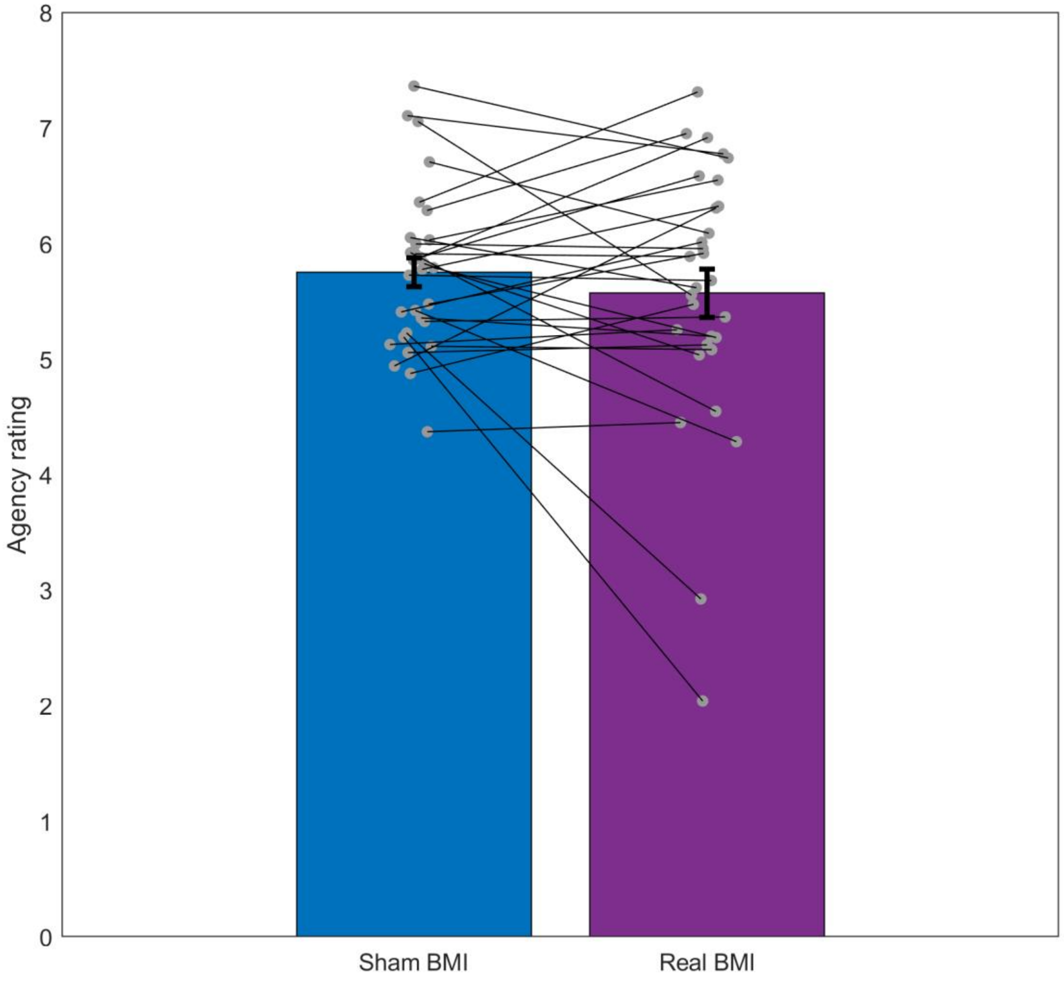

Figure S9. Comparison of mean agency ratings for each participant between sham BMI (66% of trials, used for the analysis) and real BMI (33% of trials). A t-test revealed no significant difference between the two conditions ($t(29) = 0.99$, $p = 0.33$). Error bars indicate standard errors.

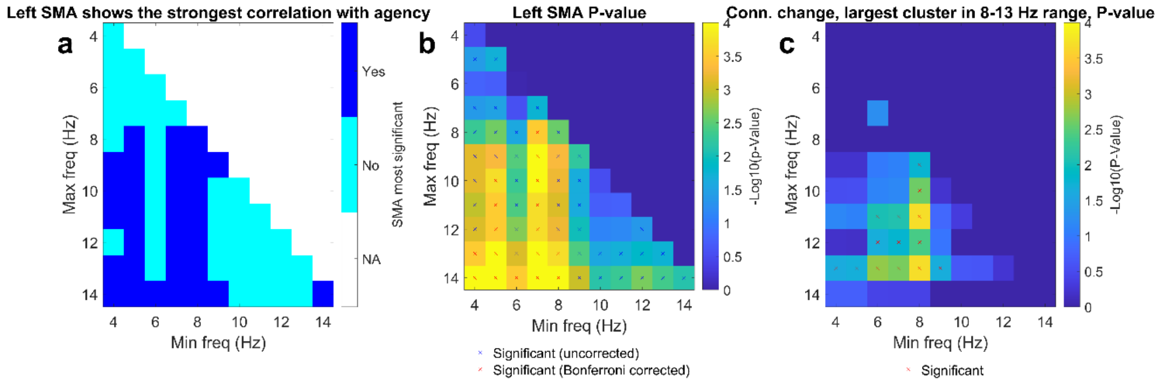

Figure S10. Robustness checks on the choice of the 8-13 Hz frequency range in Experiment 3. Panel (a) shows in dark blue the frequency ranges for which the left SMA shows the most significant phase opposition effect across all 114 ROIs, depending on the minimum (x axis) and maximum (y axis) frequency chosen for the analysis. This is the case for almost all frequency ranges covering the canonical alpha band. Panel (b) shows the uncorrected log p-value for the left SMA phase opposition, again depending on the chosen frequency range. Blue crosses denote a significant uncorrected p-value, red crosses denote a significant p-value after Bonferroni correction across 114 ROIs. Panel (c) shows the log p-value for the largest cluster of connectivity increase in optimal phase trials (as in Fig. 7c) overlapping with the alpha band (8-13 Hz), as a function of the frequency range chosen to determine the individual optimal phase. Red crosses indicate significant clusters of connectivity change.

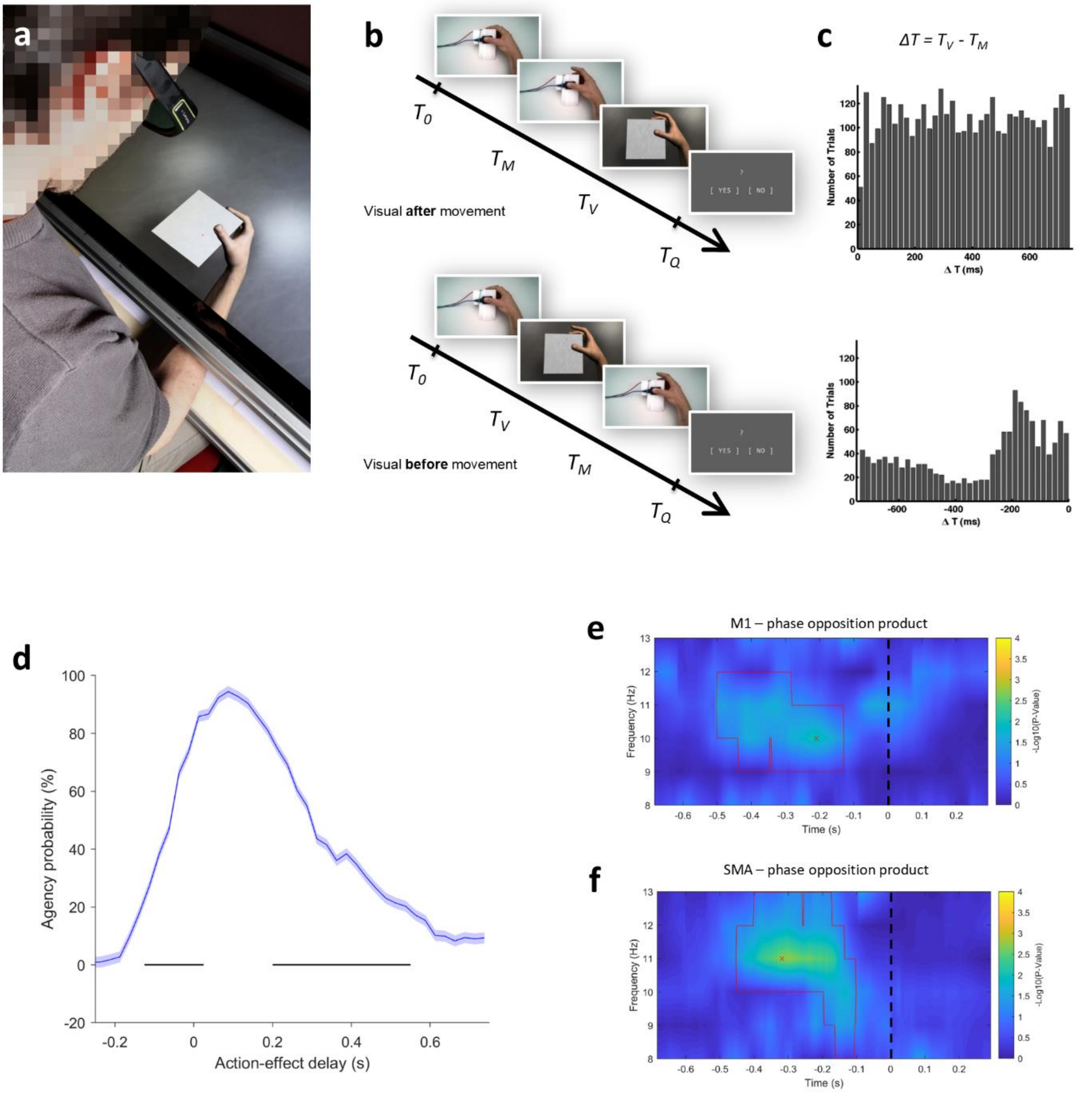

Figure S11. experiment 4 reproducing the main finding of Experiment 3 with an alternative paradigm. (a) Ten participants sat at a table and saw a stereoscopic virtual hand co-localized with their (occluded) real hand. Participants performed voluntary finger deflections and saw visual consequences (animated finger lift). Using a haptic sensor and predictive algorithms, some trials resulted in movement onsets preceding the visual consequences (positive delay) and others where the visual consequences preceded the movement (negative delay). After each trial participants answered whether or not the movement, they saw corresponded to the movement they made. (b) The two possible orders of events. The visual event occurs after the motor action (case 1), or the visual event occurs before the motor action (case 2). Case 1 corresponds to a positive ΔT ($T_V > T_M$). It is simulated by simply adding a delay after the detection of the movement onset, thus allowing the system to distribute the ΔT uniformly in the range [0, 750ms]. Case 2 corresponds to a negative ΔT ($T_M > T_V$). It is simulated by launching the animation before movement onset at a pre-determined

time, not knowing when the subject was going to perform the action: to maximise the chances of provoking some T_V close to T_M, where interesting effects are expected, the system computed the probability of the next T_M based on past trials, and targets a uniform distribution of ΔT in the range [-750, 0ms]. (c) Actual distribution of obtained action-effect delays for case 1 and case 2 trials. (d) Average agency percentage across participants, as a function of the action- effect delay. Shades indicate standard errors. The region with delay < -250 ms is not shown, as agency ratings were constantly at 0. The black horizontal lines indicate regions where the average agency probability is between 20 and 80 %, used in subsequent analyses. (e) Negative log p-values (from comparison with 10000 permutations) for the phase opposition product for the left M1 contrasting positive and negative agency trials in the region where average agency was between 20 and 80 %. We performed the analysis on these delays, eliciting uncertain agency, in order to maximise the contribution of endogenous oscillations, excluding regions where agency ratings were constantly positive or negative. In the -0.5/0 s, 8/13 Hz region, the p-value was 0.027. The red contour denotes a significant cluster of time-frequency (p = 0.037). (f) Results of the same analysis in the left SMA. In the -0.5/0 s, 8/13 Hz region, the p-value was 0.0055. The red contour denotes a significant cluster of time-frequency (p = 0.013).

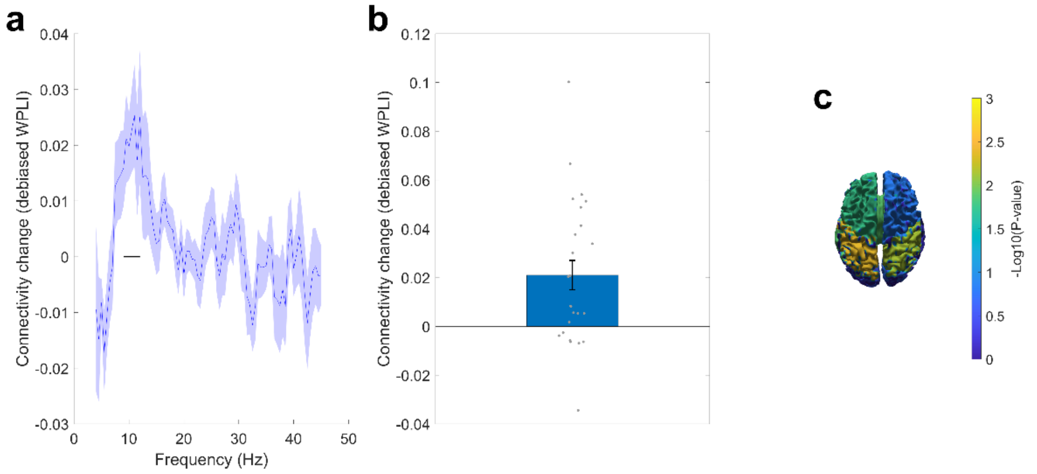

Figure S12. Results of the connectivity analysis using left M1 as a seed. (a) Average connectivity changes from the left SMA to all other cortical regions, computed in the 0.2-1.2 seconds window from movement onset, when contrasting trials in which the movement started in the optimal vs. non-optimal alpha oscillatory phase. The black line indicates the cluster of frequencies (9-12 Hz) in which significant changes occur after correcting for multiple comparisons. The cluster survives multiple comparison correction (p = 0.019). Shades indicate standard errors. (b) Connectivity change in the 9-12 Hz range presented individually for all the analysed subjects (N = 25). The connectivity change is significant at p = 0.0019. The error bar indicates the standard error. (c) Map of connectivity change log p-values on eight cerebral macro-regions. Four regions show significant changes (t-test, two

tailed): parietal left (uncorrected p = 0.0041), parietal right (uncorrected p = 0.0073), temporal left (uncorrected p = 0.0130), frontal left (uncorrected p = 0.0175).

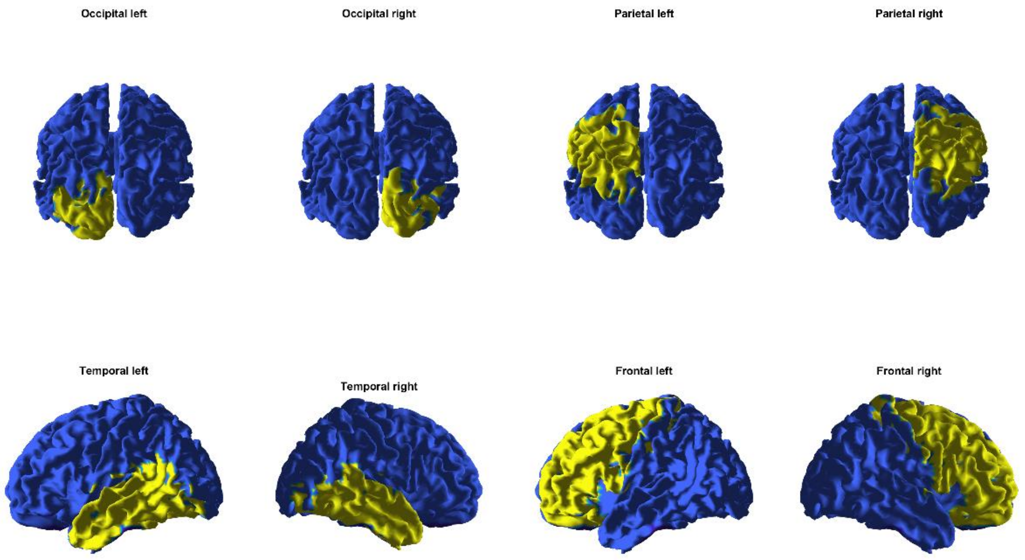

Figure S13. Representation of the subdivision in 8 macro-regions for the connectivity analysis, based on cerebral lobe and hemisphere.

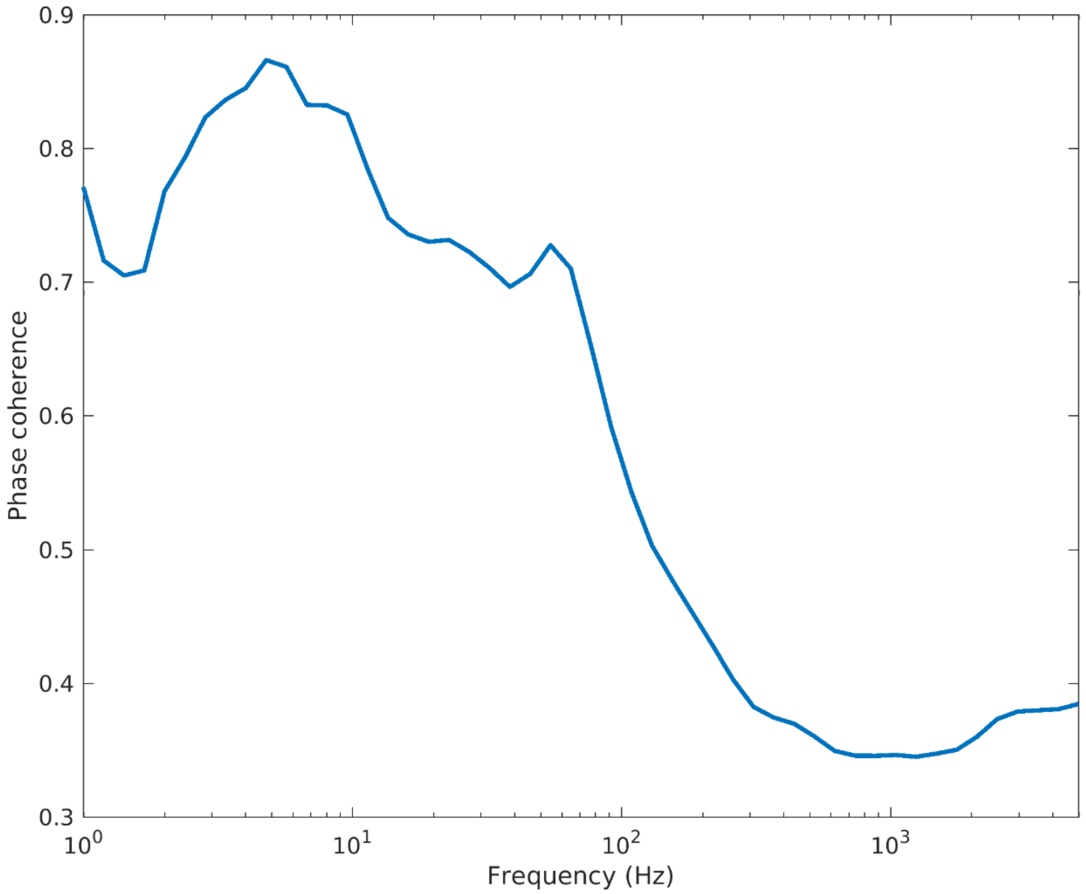

Figure S14. Phase coherence across channels in the intracranial setup. The phase coherence was computed as the average ITC (formula (3) of the main text) across channels over a 15 minutes sample recording (raw data after artefact blanking), for frequencies between 1 Hz and 5 KHz. In the theta-alpha range, the ITC is consistently above 0.8, indicating small loss of information in this frequency band after averaging across channels.

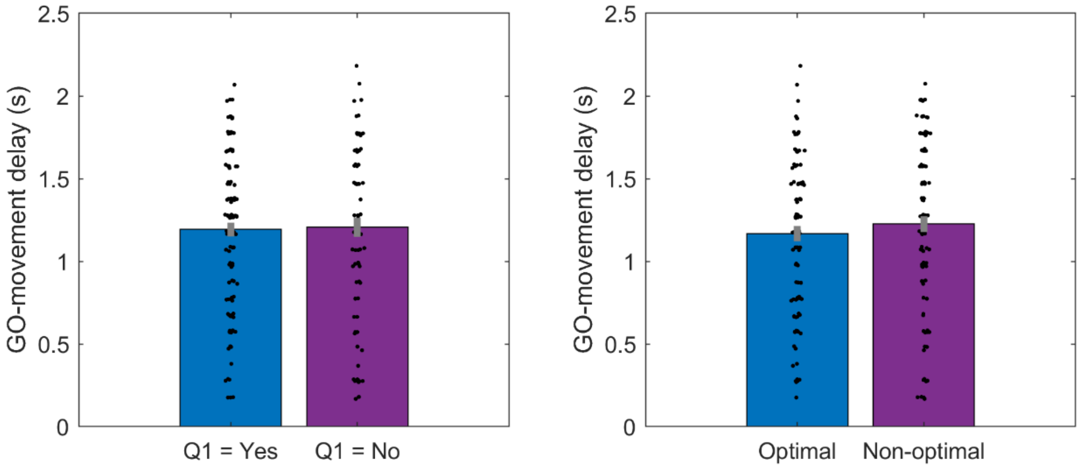

Figure S15. Control analyses on the decoder delays. The left panel shows the delay between go cue and NMES movement onset compared for high and low agency trials (t-test, two tailed, $p = 0.84$). The right panel compares the same delay between trials starting in the optimal vs. non-optimal phase (t-test, two tailed, $p = 0.48$). The grey bars represent standard errors.

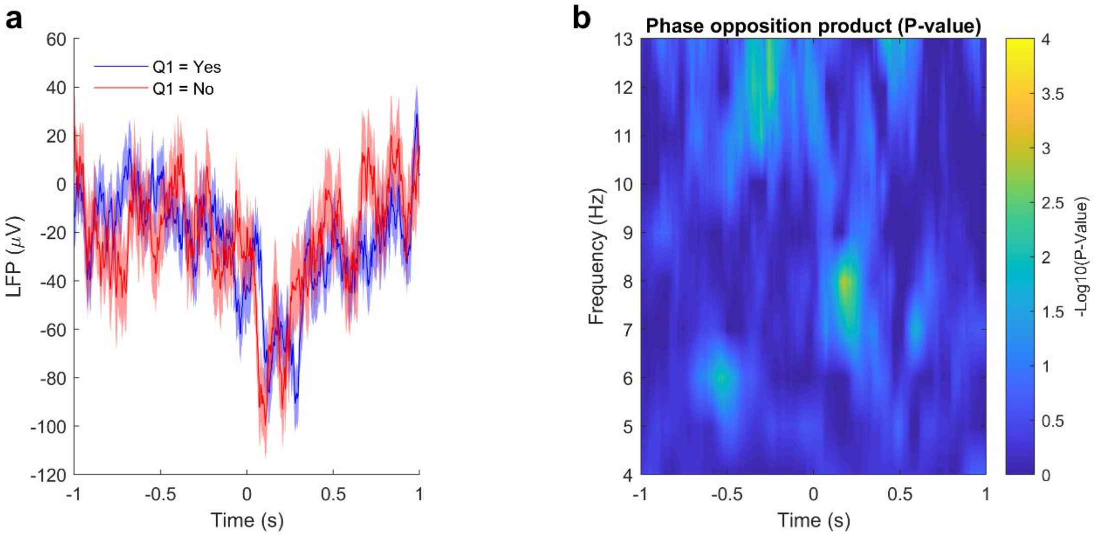

Figure S16. Phase opposition analysis for Experiment 1, time-locked to a simple threshold on M1 firing rates. Firing rates were computed on a symmetrical window of 2 seconds around the movement, and normalized by dividing them by the average firing rate on the first 100 ms bin. Normalization was required to account for intersession variability. Then, the threshold was determined as the median normalized firing rate at movement onset. Our analysis was then time-locked to the first 100 ms bin to reach such threshold. Panel (a) shows the average LFP for high and low agency trials time-locked to the firing threshold, instead of the actual movement onset as in the main text. Panel (b) shows uncorrected log p-values for the phase opposition analysis (comparison with 10000 permutations, one tailed) on the same data. No cluster survived multiple comparison correction ($p > 0.33$).

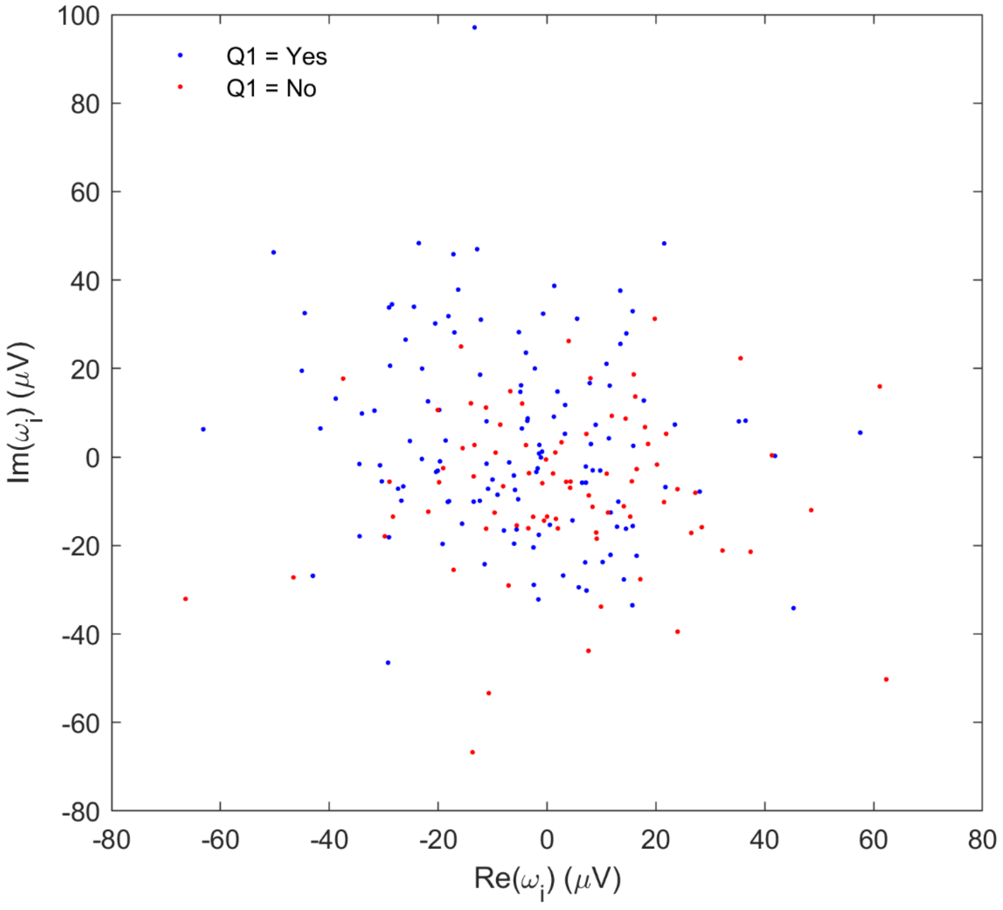

Figure S17. Contribution of LFP amplitude and phase. Real (x axis) and imaginary (y axis) part of analytic signals, corresponding to amplitude and phase of the 8 Hz component of the LFP at -256 ms, for individual trials in Experiment 1, extracted through Morelet wavelets.

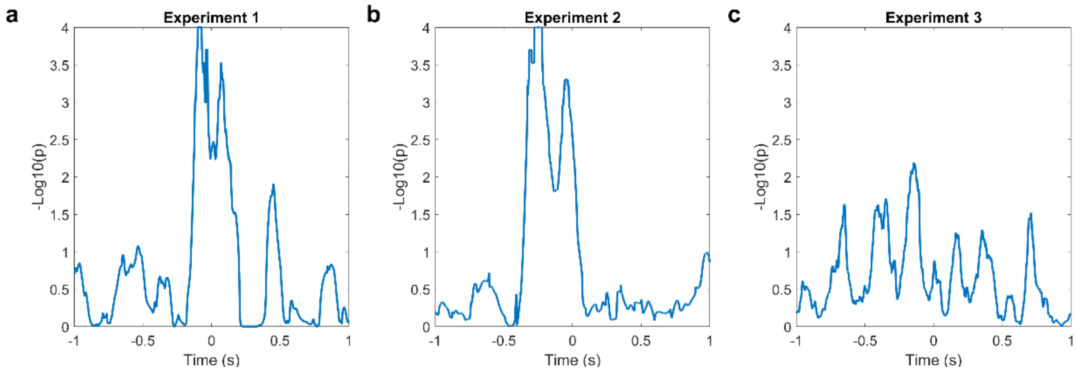

Figure S18. Replication of the main effect of phase on the sense of agency using a causal filter for Experiment 1 (a), Experiment 2 (b) and Experiment 3 (c). The plots show the uncorrected log p-values for the phase opposition product computed on phase angles extracted by applying the Hilbert transform on data filtered using a causal filter, to demonstrate that the effect genuinely starts before movement onset. Data for experiments 1 and 2 was filtered in the 6-10 Hz range, data for Experiment 3 was filtered in the 8-13 Hz range and the signal was taken from the left SMA. P-values were obtained by comparing true phase opposition values to 10000 permutations of the individual trial labels. Replicating the analysis presented for Experiment 3, by averaging phase opposition values in the -0.5/0s range, the uncorrected p-value is .0052.

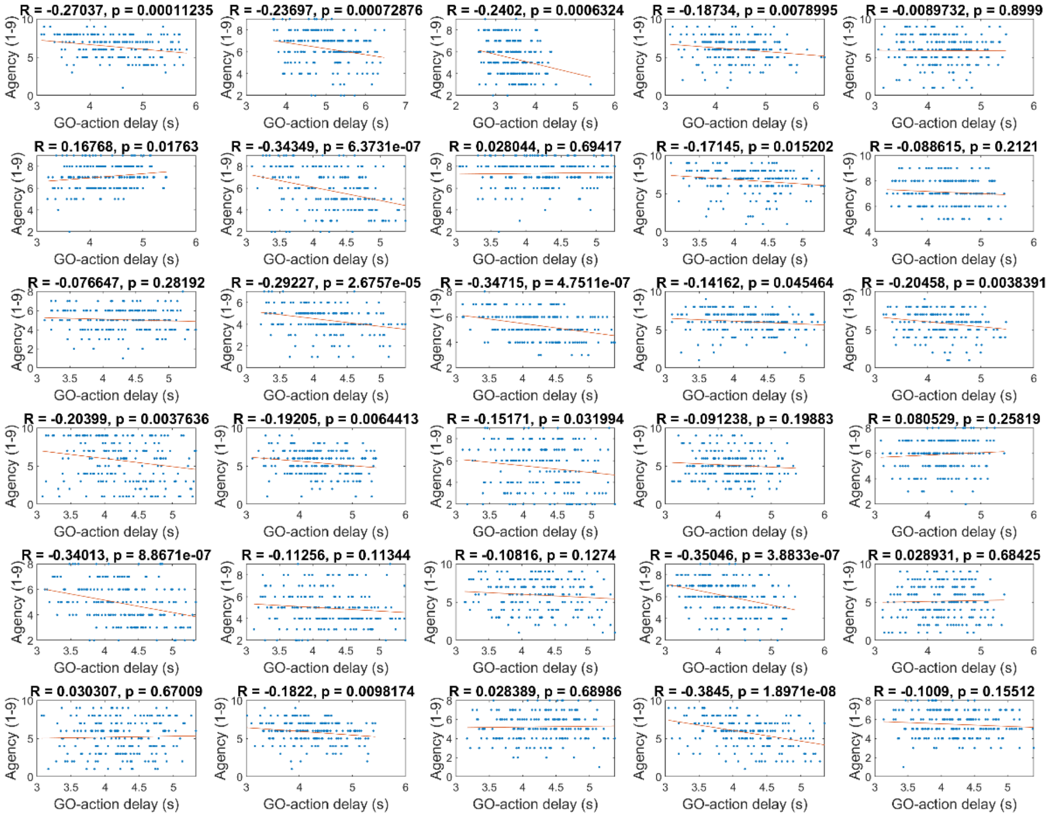

Figure S19. Correlation between agency ratings and the randomized delay between go cue and hand movement in the 30 subjects of Experiment 3.

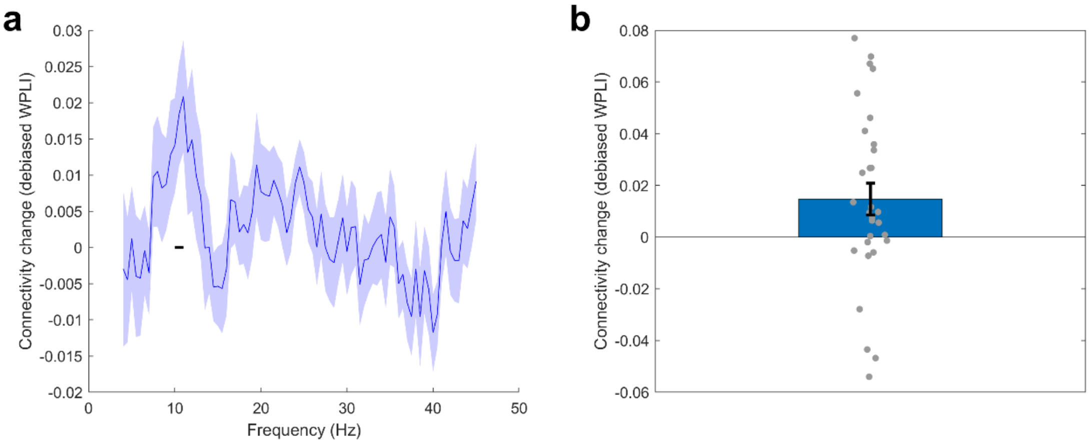

Figure S20. results of the connectivity analysis performed on all 30 participants, without excluding the 5 participants without a significant phase-agency modulation. (a) Average connectivity changes from the left SMA to all other cortical regions, computed in the 0.2-1.2 seconds window from movement onset, when contrasting trials in which the movement started in the optimal vs. non-optimal alpha oscillatory phase. The black line indicates the cluster of frequencies (9-12 Hz) in which significant changes occur, without correcting for multiple comparisons. The cluster does not survive multiple comparison correction (p = 0.12). Shades indicate standard errors. (b) Connectivity changes in the 9-12 Hz range presented individually for all the analysed subjects. The connectivity changes are significant after a two tailed t-test at p = 0.024. The error bar indicates the standard error.

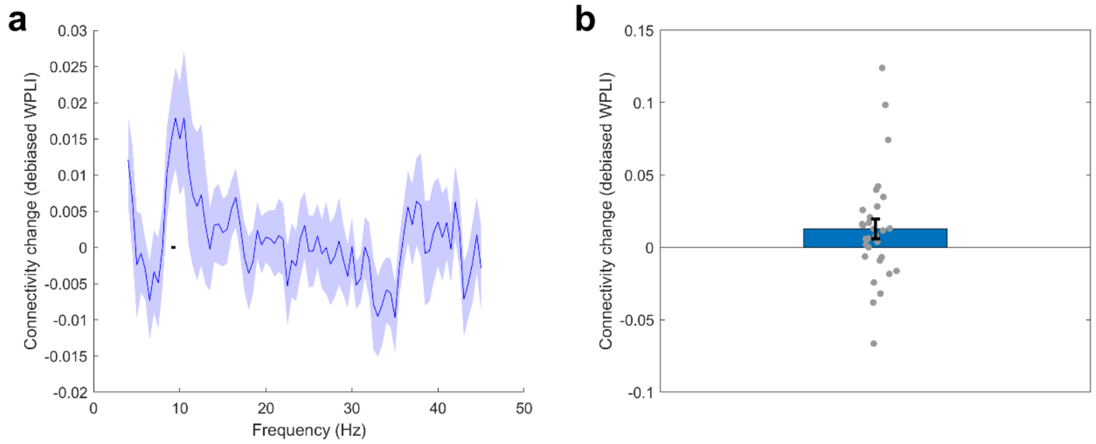

Figure S21. Results of the connectivity analysis performed on all 30 participants and using the same optimal phase angle for all subjects. The phase angle was computed on the group level highest phase opposition time-frequency point in the left SMA (9 Hz, -304 ms). (a) Average connectivity changes from the left SMA to all other cortical regions, computed in the 0.2-1.2 seconds window from movement onset, when contrasting trials in which the movement started in the optimal vs. non-optimal alpha oscillatory phase. The black line indicates frequencies in which significant changes occur, without correcting for multiple comparisons (9-10 Hz). Shades indicate standard errors. (b) Connectivity changes in the 9-12 Hz range presented individually for all the analysed subjects. The connectivity change is significant if using a one-way t-test, p = 0.038. The error bar indicates the standard error.

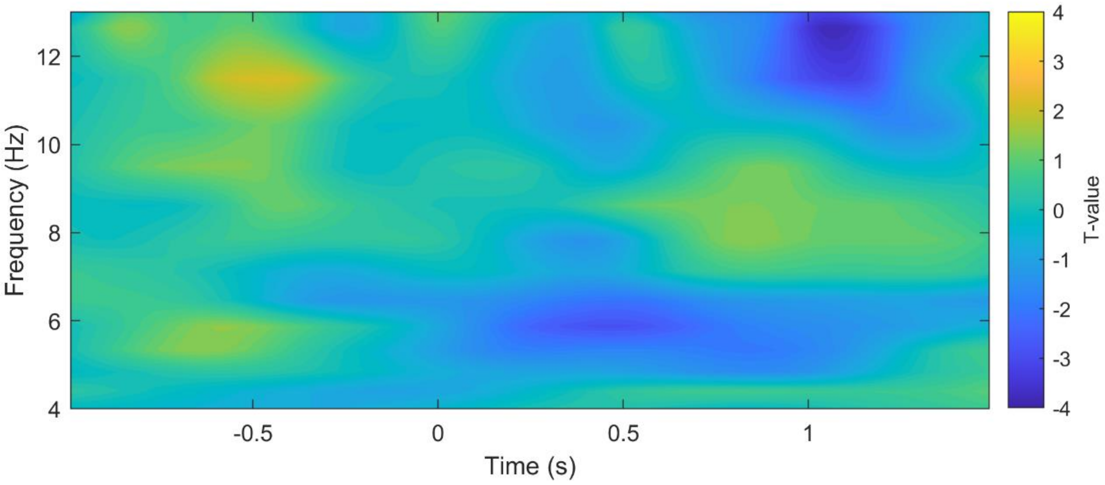

Figure S22. Control analysis of oscillatory power as a function of optimal vs. non-optimal phase for Experiment 3, power in the left SMA is displayed. Power decreases slightly and not significantly (two tailed t-test, p = 0.33) in the time-frequency range used for connectivity (9-12 Hz, 0.2-1.2 s), ruling out the possibility that the observed connectivity changes are due to changes in oscillatory power.

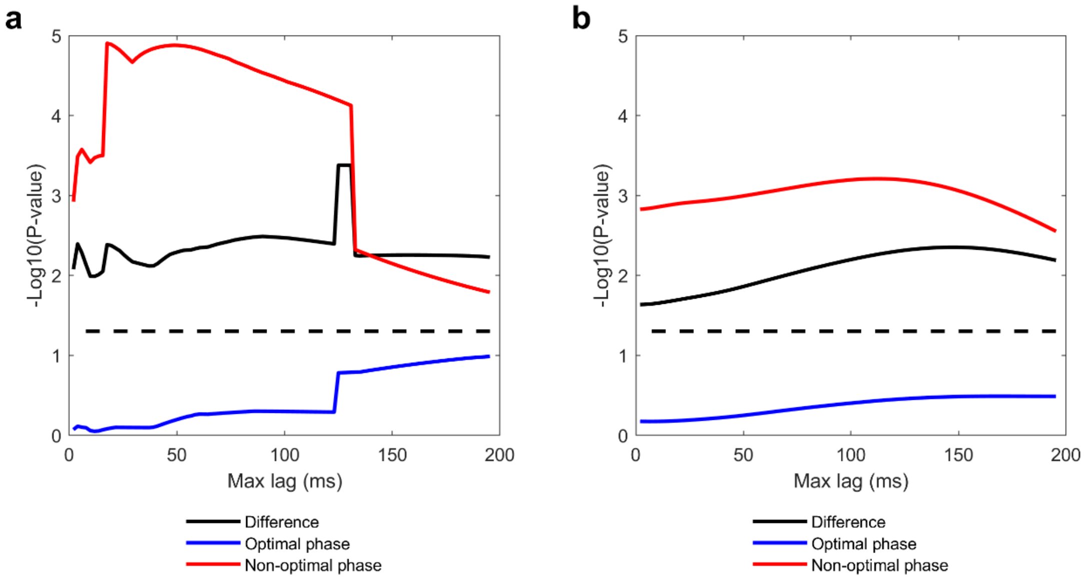

Figure S23. Sensitivity to the maximal lag used in the connectivity directionality analysis. Panel (a) shows the results of the directionality analysis presented in the main text, comparing the peak of the delayed phase coherence in optimal and non-optimal trials, as a function of the maximal time lag used when extracting the peak. The red curve shows the uncorrected log p-values (t-test, two tailed) when comparing the peak (positive, or to SMA, on average at all lags) in the non-optimal phase to 0. The blue curve shows the uncorrected log p-value when comparing the peak (negative, or from SMA, on average at all lags) in the optimal phase to 0. The black curve shows the uncorrected log p-values when comparing the peaks across the two conditions, as in panel 7e of the main text. The analysis is significant at all considered maximum lags. Panel (b) shows an alternative analysis, comparing connectivity at positive lags to connectivity at negative lags through a paired t-test. The blue and red curves show again the analysis within the optimal and non-optimal conditions, and the black curve shows the comparison of the two conditions. The direction of the effect is coherent with panel (a), with the optimal phase connectivity being slightly, but not significantly directed from SMA, and significantly directed towards SMA in the non-optimal phase.
