# Supplementary material for: Pre-movement sensorimotor oscillations shape the sense of agency by gating cortical connectivity
Source: Nat Commun. 2025 Apr 16;16:3594. doi: 10.1038/s41467-025-58683-9 (PMC12000325; doi:10.1038/s41467-025-58683-9)
Supplement: Supplementary file 2 — Reporting Summary [file 41467_2025_58683_MOESM2_ESM.pdf]

nature portfolio

Corresponding author(s): Tommaso Bertoni

Last updated by author(s): Oct 7, 2024

Reporting Summary

Nature Portfolio wishes to improve the reproducibility of the work that we publish. This form provides structure for consistency and transparency in reporting. For further information on Nature Portfolio policies, see our Editorial Policies and the Editorial Policy Checklist.

Statistics

For all statistical analyses, confirm that the following items are present in the figure legend, table legend, main text, or Methods section.

n/a	Confirmed	
☐	☒	The exact sample size (*n*) for each experimental group/condition, given as a discrete number and unit of measurement
☐	☒	A statement on whether measurements were taken from distinct samples or whether the same sample was measured repeatedly
☐	☒	The statistical test(s) used AND whether they are one- or two-sided<br>*Only common tests should be described solely by name; describe more complex techniques in the Methods section.*
☐	☒	A description of all covariates tested
☐	☒	A description of any assumptions or corrections, such as tests of normality and adjustment for multiple comparisons
☐	☒	A full description of the statistical parameters including central tendency (e.g. means) or other basic estimates (e.g. regression coefficient) AND variation (e.g. standard deviation) or associated estimates of uncertainty (e.g. confidence intervals)
☐	☒	For null hypothesis testing, the test statistic (e.g. *F*, *t*, *r*) with confidence intervals, effect sizes, degrees of freedom and *P* value noted<br>*Give P values as exact values whenever suitable.*
☒	☐	For Bayesian analysis, information on the choice of priors and Markov chain Monte Carlo settings
☒	☐	For hierarchical and complex designs, identification of the appropriate level for tests and full reporting of outcomes
☐	☒	Estimates of effect sizes (e.g. Cohen's *d*, Pearson's *r*), indicating how they were calculated

Our web collection on statistics for biologists contains articles on many of the points above.

Software and code

Policy information about availability of computer code

Data collection	Data from the implanted participant was collected through the Neuroport neural data acquisition system. The online decoding of intended movements was performed with custom made code running on MATLAB (version 2012a). Data from healthy controls was collected using custom code in Python and the Neurodecode Python-based platform (https://github.com/dbdq/neurodecode)
Data analysis	Data analysis was conducted in MATLAB (version 2022a).

For manuscripts utilizing custom algorithms or software that are central to the research but not yet described in published literature, software must be made available to editors and reviewers. We strongly encourage code deposition in a community repository (e.g. GitHub). See the Nature Portfolio guidelines for submitting code & software for further information.

Data

Policy information about availability of data

All manuscripts must include a data availability statement. This statement should provide the following information, where applicable:

- Accession codes, unique identifiers, or web links for publicly available datasets
- A description of any restrictions on data availability
- For clinical datasets or third party data, please ensure that the statement adheres to our policy

Data from the implanted participant cannot be made available due to privacy reasons. Pre-processed EEG data is available at https://osf.io/4w3t6/?view_only=c5c108ae37304e8f9ed9a9aad61bd763.

Research involving human participants, their data, or biological material

Policy information about studies with human participants or human data. See also policy information about sex, gender (identity/presentation), and sexual orientation and race, ethnicity and racism.

Reporting on sex and gender	Sex data was collected based on self reports. It is reported for completeness in the methods section, but no sex-based analysis was conducted, as there is no reason to expect sex-specific effects in our investigation of low-level neurophysiological correlates of the sense of agency.
Reporting on race, ethnicity, or other socially relevant groupings	No data on race or ethnicity was collected, as there is no reason to expect these aspects to affect the low level neural phenomena investigated in this study.
Population characteristics	See above
Recruitment	The implanted participant was enrolled in a pilot clinical trial (NCT01997125, November 22, 2013). Healthy participants were recruited through the University of Lausanne Sona Systems platform (https://epflunil.sona-systems.com/).
Ethics oversight	The implanted participant was enrolled in a pilot clinical trial (NCT01997125, November 22, 2013). Approval for this study was obtained from the US Food and Drug Administration (Investigational Device Exemption) and The Ohio State University Medical Center Institutional Review Board (Columbus, Ohio). Approval for the experiment on healthy controls was granted by the ethical committee of the Vaud canton, Switzerland.

Note that full information on the approval of the study protocol must also be provided in the manuscript.

Field-specific reporting

Please select the one below that is the best fit for your research. If you are not sure, read the appropriate sections before making your selection.

☒ Life sciences ☐ Behavioural & social sciences ☐ Ecological, evolutionary & environmental sciences

For a reference copy of the document with all sections, see nature.com/documents/nr-reporting-summary-flat.pdf

Life sciences study design

All studies must disclose on these points even when the disclosure is negative.

Sample size	Two out of four experiments were performed on one unique implanted user of an invasive BMI system. The sample size for the cohort of healthy controls (N = 30) was based on previous studies investigating the perceptual role of pre-stimulus neural oscillations (e.g., Busch et al., JNeurosci, 2009). The fourth experiment recruited 10 participants to confirm results from the third experiment.
Data exclusions	Bad epochs/channels/independent components were removed from the EEG data as described in the methods, following standard practices in EEG analysis.
Replication	A cohort of 30 participants was recruited to replicate the result observed in one implanted participant. A further cohort of 10 participants was recruited to confirm these results with a different paradigm.
Randomization	Participants were not allocated to different cohorts, as the analyses supporting our results are based on subjective reports collected within participants. No randomization was therefore needed.
Blinding	The analyses performed are based on objective and reproducible methods and therefore do not require blinding.

Reporting for specific materials, systems and methods

We require information from authors about some types of materials, experimental systems and methods used in many studies. Here, indicate whether each material, system or method listed is relevant to your study. If you are not sure if a list item applies to your research, read the appropriate section before selecting a response.

Materials & experimental systems

n/a	Involved in the study
☒	☐ Antibodies
☒	☐ Eukaryotic cell lines
☒	☐ Palaeontology and archaeology
☒	☐ Animals and other organisms
☒	☐ Clinical data
☒	☐ Dual use research of concern
☒	☐ Plants

Methods

n/a	Involved in the study
☒	☐ ChIP-seq
☒	☐ Flow cytometry
☒	☐ MRI-based neuroimaging

Plants

Seed stocks	*Report on the source of all seed stocks or other plant material used. If applicable, state the seed stock centre and catalogue number. If plant specimens were collected from the field, describe the collection location, date and sampling procedures.*
Novel plant genotypes	*Describe the methods by which all novel plant genotypes were produced. This includes those generated by transgenic approaches, gene editing, chemical/radiation-based mutagenesis and hybridization. For transgenic lines, describe the transformation method, the number of independent lines analyzed and the generation upon which experiments were performed. For gene-edited lines, describe the editor used, the endogenous sequence targeted for editing, the targeting guide RNA sequence (if applicable) and how the editor was applied.*
Authentication	*Describe any authentication procedures for each seed stock used or novel genotype generated. Describe any experiments used to assess the effect of a mutation and, where applicable, how potential secondary effects (e.g. second site T-DNA insertions, mosiacism, off-target gene editing) were examined.*
